# P-2070. Evaluation of Human Immunodeficiency Virus (HIV) Virologic Suppression among Reincarcerated Individuals within in the Illinois Department of Corrections

**DOI:** 10.1093/ofid/ofaf695.2234

**Published:** 2026-01-11

**Authors:** Hadeel Fouad, Melissa E Badowski, Joy Lee, Jennifer Flores, Jane Park, Mahesh C Patel, Brian W Drummond, Scott Borgetti, Drew Halbur, Emily N Drwiega

**Affiliations:** University of Illinois at Chicago College of Pharmacy, Park Ridge, IL; University of Illinois Chicago, Chicago, Illinois; UIC, Park Ridge, Illinois; UIC, Park Ridge, Illinois; UIC, Park Ridge, Illinois; University of Illinois Chicago, Chicago, Illinois; Telemedicine Team Member, Chicago, IL; University of Illinois at Chicago, Chicago, Illinois; Walgreens Pharmacy, Chicago, Illinois; University of Illinois Chicago, Chicago, Illinois

## Abstract

**Background:**

The University of Illinois Hospital and Health Sciences Center (UIH) multidisciplinary telemedicine clinic provides HIV care to justice-involved individuals in the Illinois Department of Corrections (IDOC). Post-release HIV care remains challenging, with virologic suppression (VS) dropping from 73% at release to 49.7% at reincarceration into IDOC in a prior study. To improve continuity of care, wrap-around services were expanded from follow-up care at UIH to medical insurance assistance, case management, employment/housing support, and statewide follow-up care.

**Figure:**
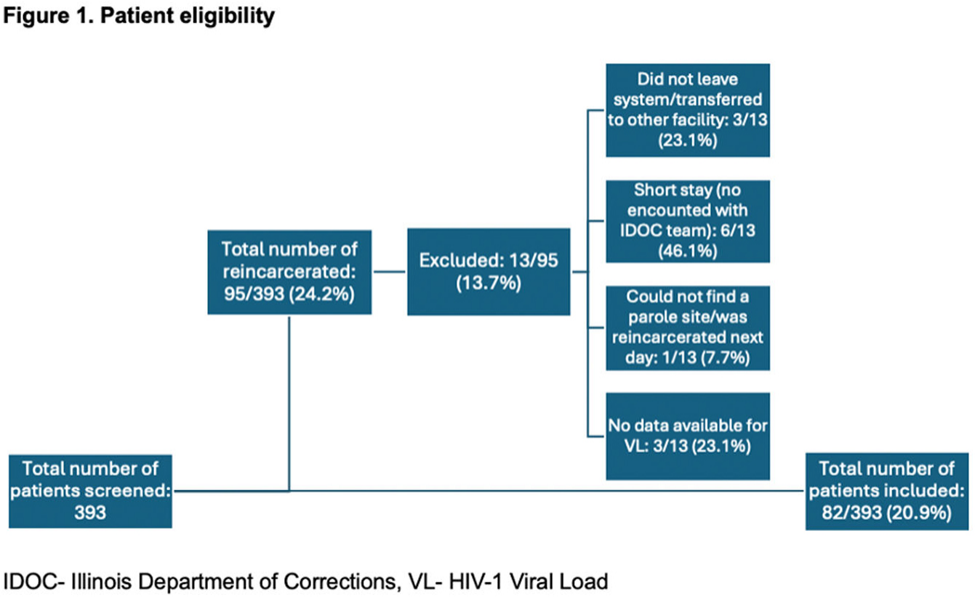

**Figure:**
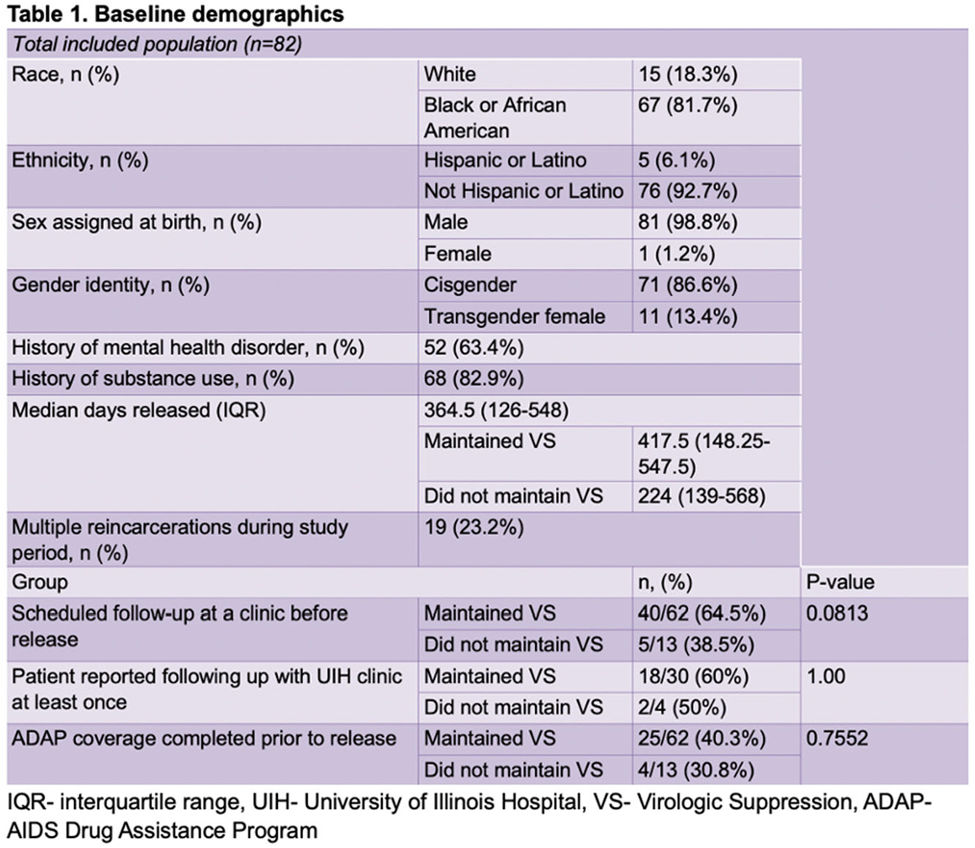

**Methods:**

This IRB-approved, retrospective cohort study taking place from 1/1/21- 8/31/24 analyzed demographic, clinical, and social determinants of health in people with HIV aged ≥18 years, in custody in IDOC, released and reincarcerated during the study period. The primary outcome was a change in the number of patients with complete VS at time of release as compared to reincarceration. Secondary outcomes included change in immunologic function and identification of factors influencing loss of VS upon reincarceration.

**Figure:**
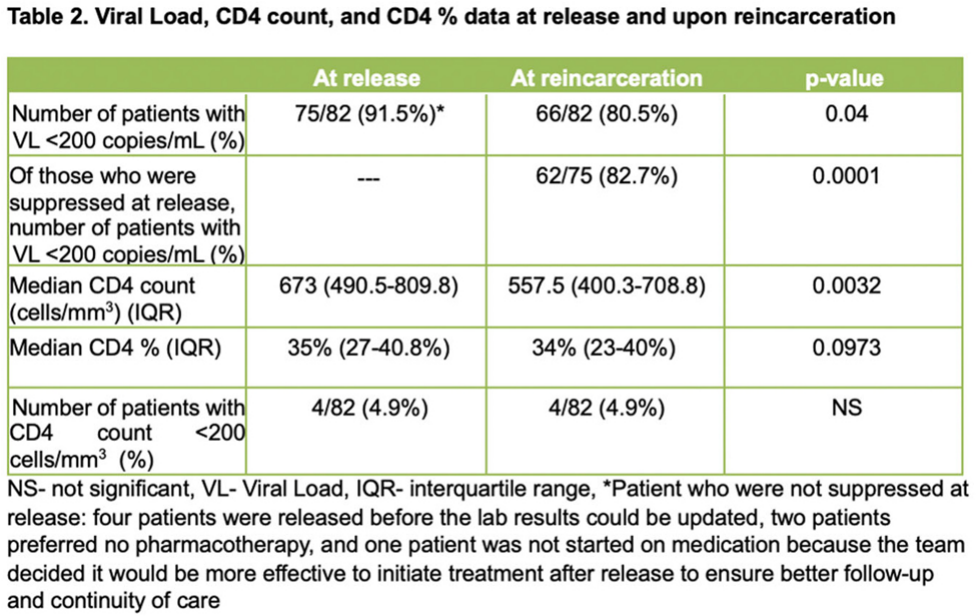

**Figure:**
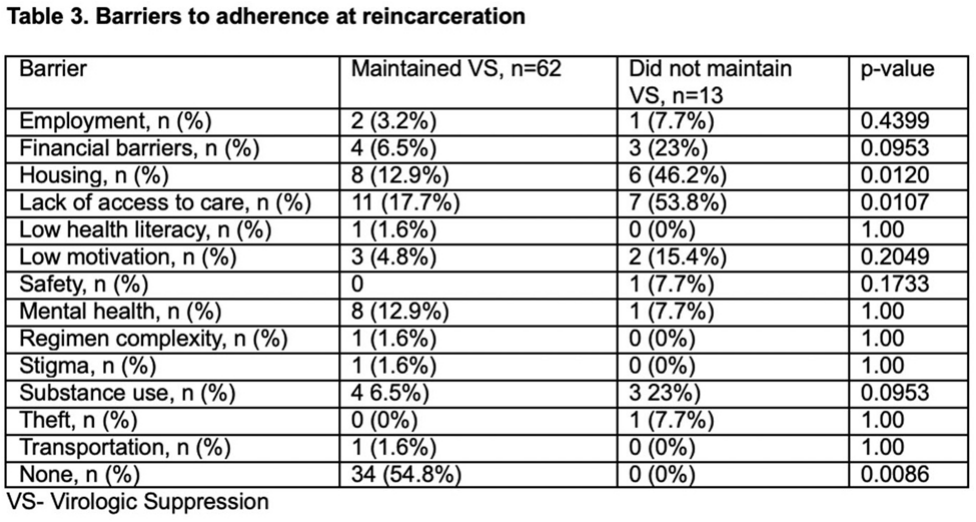

**Results:**

Of 393 patients released during the study period, 95 were reincarcerated, 82 were included, and 75 had VS at time of release. [Figure 1] Of the 75 who achieved VS at release, 62 maintained VS (group 1) at reincarceration, while 13 experienced loss of VS (group 2) (*p = 0.0001)*. Median CD4 count of those who met inclusion criteria declined significantly from release to reincarceration (p = 0.0032) but the change in CD4% was not significant (p = 0.0973).

No difference was found between group 1 and 2 in scheduling a statewide follow-up visit (p = 0.0813), following up at UIH clinic at least once (p = 1.00), or having ADAP coverage (*p = 0.7552*). Loss of VS was significantly associated with patient-reported housing instability (*p = 0.0046*), lack of access to care (*p = 0.0056*), living outside of Chicago (*p = 0.0318*) and transferring from a jail in a rural setting (*p = 0.0054*).

**Conclusion:**

As the role of IDOC telemedicine team expanded, the proportion of individuals who maintained VS upon reincarceration increased from 49.7% in 2014 to 82.7% in 2024. While interventions show progress, more targeted efforts are needed to address housing, care access, and non-Chicago residency.

**Disclosures:**

Scott Borgetti, MD, GlaxoSmithKline: Grant/Research Support

